# Assessing Public Opinion on CRISPR-Cas9: Combining Crowdsourcing and Deep Learning

**DOI:** 10.2196/17830

**Published:** 2020-08-31

**Authors:** Martin Müller, Manuel Schneider, Marcel Salathé, Effy Vayena

**Affiliations:** 1 Digital Epidemiology Lab School of Life Sciences, School of Computer and Communication Sciences EPFL Geneva Switzerland; 2 Health Ethics and Policy Lab Department of Health Sciences and Technology ETH Zurich Zurich Switzerland

**Keywords:** CRISPR, natural language processing, sentiment analysis, digital methods, infodemiology, infoveillace, empirical bioethics, social media

## Abstract

**Background:**

The discovery of the CRISPR-Cas9–based gene editing method has opened unprecedented new potential for biological and medical engineering, sparking a growing public debate on both the potential and dangers of CRISPR applications. Given the speed of technology development and the almost instantaneous global spread of news, it is important to follow evolving debates without much delay and in sufficient detail, as certain events may have a major long-term impact on public opinion and later influence policy decisions.

**Objective:**

Social media networks such as Twitter have shown to be major drivers of news dissemination and public discourse. They provide a vast amount of semistructured data in almost real-time and give direct access to the content of the conversations. We can now mine and analyze such data quickly because of recent developments in machine learning and natural language processing.

**Methods:**

Here, we used Bidirectional Encoder Representations from Transformers (BERT), an attention-based transformer model, in combination with statistical methods to analyze the entirety of all tweets ever published on CRISPR since the publication of the first gene editing application in 2013.

**Results:**

We show that the mean sentiment of tweets was initially very positive, but began to decrease over time, and that this decline was driven by rare peaks of strong negative sentiments. Due to the high temporal resolution of the data, we were able to associate these peaks with specific events and to observe how trending topics changed over time.

**Conclusions:**

Overall, this type of analysis can provide valuable and complementary insights into ongoing public debates, extending the traditional empirical bioethics toolset.

## Introduction

Genome editing has many potential applications, ranging from gene therapy [1] to crop enhancement [2] and production of biomolecules [3,4]. While it has been possible to modify the genomes of eukaryotic cells since the 1980s, traditional methods have proven to be rather impractical, inaccurate, or impossible to use at scale [5-8]. Accurately targeted gene editing has only become possible within the last decade [9,10] using a CRISPR-Cas9–based method. In 2013, the method was further developed to be used on human cells [11,12], which allowed for the first successful experiment to alter the human germline DNA of non-viable embryos in April 2015 [13]. The experiment, conducted by a group of Chinese scientists, raised ethical concerns among researchers and the general public about the potential far-reaching consequences of introducing germline modifications [14,15]. Such ethical concerns include unexpected side effects on the evolution of humans, as well as cultural and religious arguments. In November 2018, Jiankui He announced the genetic editing of two viable human embryos with the goal of introducing HIV resistance [16]. The work came to be known to a global public under the term “CRISPR babies” and was condemned by the scientific community as unethical, unnecessary, and harmful to the two babies [17,18].

As the costs of the technology drop further and usage becomes more widespread, governments and policy makers are faced with the challenging task of posing adequate ethical restrictions to prevent misuse. To gain time to introduce appropriate ethical frameworks, some scientists have called for a moratorium on genetically editing the human germline [19-21]. Previous studies on opinion towards GMO plants highlight how certain events or scandals (eg, with respect to food safety) may have a major long-term impact on public opinion and later drive policy decisions [22-25]. Understanding the public attitudes towards topics such as CRISPR is therefore of paramount importance for policy making [26,27].

Several surveys have been conducted with the goal of evaluating the public’s perception of CRISPR and genetic engineering in general [28-32]. Such surveys have found that participants are largely in favor of the technology used for somatic purposes (eg, in the context of treatment) but less so for germline editing, especially if this is not for clearly medical purposes. Additionally, the studies underline certain demographic correlations (eg, that women, people belonging to ethnic minorities, and religious communities are more critical about the potential applications of CRISPR [28,30]). Somewhat unsurprisingly, the surveys also show that public views are not always aligned with expert opinions [32]. A recent study that explored coverage of news articles on CRISPR in North America between 2012 and 2017 found CRISPR to be overwhelmingly portrayed as positive and potentially overhyped in news media compared to the public’s views [33].

Social media platforms allow people to discuss a topic online with other people around the globe, creating an abundance of semistructured conversational data. Sentiment analysis provides a way to study people’s perception of a topic, based on personal statements, and to process large volumes of such data in an automated way. Sentiment analysis has been used in the past to analyze different features such as emotions and polarity in several different contexts [34]. While traditional methods are based on linguistic expert knowledge (eg, rule-based methods), newer methods leverage machine learning, can be trained for specific contexts, and dominate traditional methods on polarity classification tasks [35]. Additionally, the supervised machine learning approaches have the advantage that the performance of a model for the specific context can be evaluated. The adaption to a specific context is particularly useful for tweets, which have a very specific, informal language [36]. Accordingly, machine learning methods have been successfully used for Twitter sentiment analysis [37,38]. Most classical supervised machine learning algorithms for text classification (such as Naive Bayes or support vector machines [SVMs]) rely on manual feature extraction. Recently, a type of semisupervised machine learning model called Bidirectional Encoder Representations from Transformers (BERT) has been introduced to natural language processing [39]. BERT models are pretrained on large corpuses of raw text and can be adapted to a target task in a process called transfer learning. BERT models are based on the transformer, a neural network architecture that has been shown to outperform previously mentioned models in most natural language processing tasks, including text classification and sentiment analysis [40,41]. BERT has also been used in top-ranking submissions in the SemEval2019 challenges on detection of hate speech and offensive language in social media data [42,43].

In this study, we conducted the first analysis of a complete dataset of all tweets about CRISPR published over a 6.5-year period. The analyzed timespan includes the first experiment of CRISPR on human cells in 2013 but also recent events, such as the first genetic editing of viable human babies in November 2018. Furthermore, we make use of recent advances in text classification models, such as BERT [39], which use semisupervised machine learning to generate a high-resolution temporal signal of the sentiment towards CRISPR over the observed timespan. By combining multiple text classification methods, we obtain results that can also be linked back to previous studies conducted with traditional methods, such as surveys.

## Methods

### Overview

**Figure 1:**
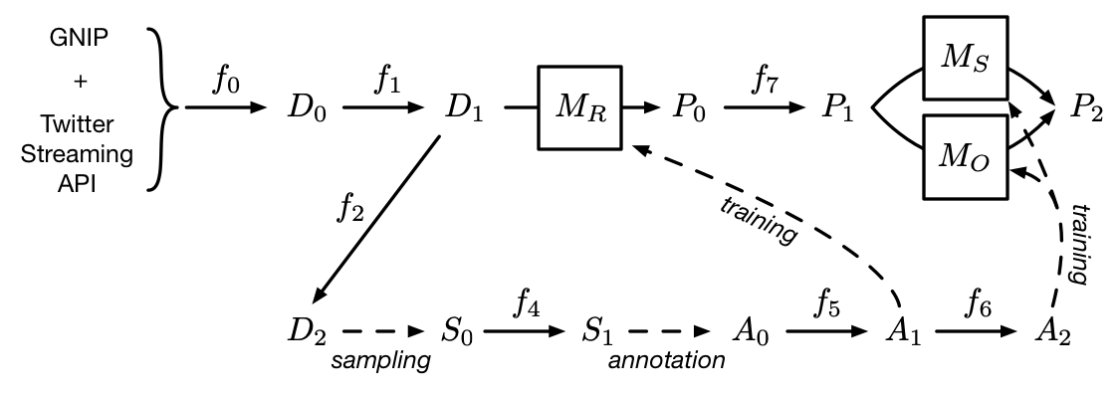
Overview of the data processing pipeline. Labels f_0-7_ denote filtering steps, D_0-2_ datasets, S_0-1_ samples, A_0-2_ annotation sets, and P_0-2_ predictions. M_R_, M_S_, and M_O_ represent machine learning models. API: application programming interface.

Our analysis consisted of 4 different explorative approaches, all of which build upon the sentiments of the tweets. Therefore, sentiment analysis represents the core of our analysis. In order to determine the sentiment for the entirety of tweets published over the last 6.5 years, we trained a predictive model on a previously manually annotated subset of the data. The process can be divided into 5 main tasks, which we describe in the following sections (see Figure 1 for an overview of the process): data collection, preparation, annotation, training, and analysis.

### Data Collection

The data set (denoted as D_0_ in Figure 1) for our analysis consists of all tweets (including retweets, quoted tweets, replies, and mentions) that match the character sequence CRISPR (in any capitalization), have been detected to be in English language, and were published between January 1, 2013 and May 31, 2019. We retrieved these data either through the Twitter Streaming API or through GNIP, a Twitter subsidiary that allows access to historical data that were not retrievable through the Twitter Streaming API. The 3 aforementioned filtering conditions were used as parameters in the retrieval through Twitter APIs (denoted as f_0_) as well as for the requested data from GNIP.

The number of tweets varied greatly over time, ranging from 4818 in 2013 to 445,744 in 2018, totaling 1,508,044 tweets by 348,502 distinct users (also refer to Multimedia Appendix 1). Since the focus was on the overall evolution of the discourse provided by aggregated information, this study considered only the text in the tweet objects and ignored user-related information (such as location) or media content (such as photos or videos). In addition, any occurrences of Twitter handles and URLs in the text were anonymized (replaced by @<user> and <url>, respectively) to protect individuals.

### Preparation

In a preparatory step, tweets suitable for annotation were selected from D_0_. As an inclusion criterion, only tweets with ≥3 English words (after removal of stop words) were considered (f_1_). Although a tweet with <3 non-stop words may express a sentiment, we chose this threshold to ensure that the annotators had at least a minimal context to determine if the tweet was in fact relevant to the topic and what sentiment it expressed. The word count was determined by the help of NLTK’s (Natural Language Toolkit, a python library for natural language processing) TweetTokenizer and English word and stop word corpora [44]. The filtering and subsequent dataset operations and analysis were carried out using pandas, a python package for data analysis [45]. The resulting dataset D_1_ (n=1,334,114) was used as the basis for the subsequent analysis. To avoid the annotation of duplicates, all retweets, quoted tweets, and other duplicates of tweets with the same text were removed, leading to dataset D_2_ (n=433,930).

Next, we selected a random sample S_0_ (n=29,238), so that we obtained a more or less evenly distributed number of tweets over the observed timespan. This was achieved by binning the data by all 77 months and selecting a constant number of tweets from each monthly bin. In contrast to a fully random sample, our sampling scheme contained no oversampling bias with regard to very recent content. Therefore, the generated sample was more representative of the whole observation period and accounted for the possibility that the nature of the tweets changed notably over time.

### Annotation

After generating the sample, the selected tweets were annotated through the Crowdbreaks platform [38,46], which uses crowdsourcing to annotate social media data. The platform allows for the creation of a question sequence that is then submitted in combination with a tweet as a task to MTurk (Amazon Mechanical Turk) [47]. The question sequence contained 3 questions for each task. The first question was on the relevance of the tweet to the topic of CRISPR-Cas9, allowing “relevant” and “not relevant” as possible answers. The second question was on the sentiment (positive, negative, or neutral), and the third question was on the organism (humans, human embryos, animals [other than human], plants, bacteria, multiple, not specified).

Before submitting the task to MTurk, the availability of the tweet was automatically checked. This was done in order to respect the user’s right to either delete their content or set it to private after the time of data collection. Filtering by tweets that were still available yielded the sample S_1_ (n=22,513), which was subsequently annotated with regard to the 3 questions mentioned earlier. This resulted in annotation set A_0_. To detect workers with questionable performance, the annotators’ raw agreement was calculated, which denotes the fraction of the number of actual agreements over the number of possible agreements an annotator had with other annotators. An annotator was considered an outlier if this value was larger than 3 standard deviations from the mean, the annotator had less than 20 possible agreements with other annotators, or the annotator was involved in less than 3 separate tasks. All annotations by outlier annotators were subsequently removed. The resulting Fleiss' kappa agreement scores [48] were 0.81 and 0.28 for the questions of relevance and sentiment, respectively. Tweets for which a unanimous consensus of at least 3 independent annotators could be found were merged into dataset A_1_. For the questions on sentiment and organism, only tweets that were labelled as relevant were considered and exported to A_2_. This resulted in 3 cleaned datasets with annotated tweets for relevance (n=16,421), sentiment (n=4718), and organism (n=1196), which we used to train 3 classifiers.

### Training

In order to classify the data with regard to relevance, sentiment, and organism, we constructed 3 classifiers: M_R_, M_S_, and M_O_, respectively. The classifiers tried to predict the respective labels from the text of the tweet alone. In the process, we analyzed the performance of 4 different classifier models: Bag of Words (BoW), Sent2Vec sentence embeddings [49] coupled with SVMs [50], FastText [51], and BERT [39]. The tokenization process was different for each model class. In order to evaluate the models, the cleaned annotation data were shuffled and split into training (80%) and test sets (20%).

For the BoW, SVM, and FastText models, we used supervised learning to train the 3 classifiers for sentiment, relevance, and organisms. A limited search of model parameters was conducted. In the case of BERT, we started from the pretrained (unsupervised) English BERT-large-uncased model provided by the Huggingface library [52] and conducted an additional step of unsupervised, domain-specific pretraining on our raw body of tweets. This model then served as the basis for the final, supervised training step (ie, fine-tuning the general model with classifier-specific labelled data). For this fine-tuning step, a learning rate of 1e-05 and 2 epochs of training were used. This work was conducted using PyTorch [53] and the Huggingface library [52].

After the training phase, we selected the classifiers for relevance, sentiment, and organism (M_R_, M_S_, and M_O_ in Figure 1) by evaluating the performance of the models on the test set (see Multimedia Appendix 2 for different model performances). The fine-tuned BERT model was the best performing sentiment classifier (M_S_), with a macro-averaged F1 score of 0.727 (F1_positive_=0.827, F1_neutral_=0.715, F1_negative_=0.639). The fine-tuned BERT model was also found to be the best performing model for the relevance (M_R_) and organism (M_O_) classifiers with macro-averaged F1 scores of 0.91 (F1_related_=0.997, F1_unrelated_=0.823) and 0.89 (F1_humans_=0.873, F1_embryos_=0.762, F1_animals_=1, F1_plants_=0.889, F1_bacteria_=0.909, F1_unspecified_=0.902), respectively.

### Prediction

For the analysis, the best performing model (fine-tuned BERT) for relevance M_R_ was used to predict dataset D_1_ and yield the predicted dataset P_0_ (n=1,334,114) of the same length containing a label for relevance. Next, all tweets predicted as not relevant were removed from P_0_, yielding the dataset P_1_ (n=1,311,544). This dataset was then used to predict sentiment and organism using the models M_S_ and M_O_, resulting in the final dataset P_2_.

### Analysis

In our analysis, we used the sentiments in relation to tweet activity (number of tweets), topics of the tweets (hashtags), organisms the tweets were talking about (predicted), and themes identified from previous studies on CRISPR mentioned earlier (through regular expressions) to gain different kinds of insights. Wherever we used sentiments for numerical calculations, we used +1 for positive, 0 for neutral, and −1 for negative sentiment. Further, we extrapolated the numbers for 2019 where applicable for better comparison since we only had data until May 31, 2019. The different parts of the analysis are explained in more detail in the following paragraphs.

The first part of the analysis was concerned with the development of the sentiment in relation to the number of tweets over time. The detection of a temporary deviation from the general sentiment was of particular interest. While we included all tweets for the analysis of activity, we excluded tweets with neutral sentiment for the analysis of sentiment to make deviations more visible. We aggregated activity and sentiments on a daily basis. For the sentiments, however, the sentiment value of a specific day was determined by taking the mean value of all positive and negative sentiments within a sliding 7-day window centered around that day (±3 days). Further, we tested whether the yearly means based on the positive and negative tweet sentiments were significantly different from each other with the Welch's *t*-test [54,55] using scipy’s statistics module [56]. We then used scipy’s module for peak detection [56] to detect events of interest, using a relative prominence cut-off of 0.2. In order to identify potential sources for the change in sentiment, we manually identified major events that relate to CRISPR.

In the second part, we used the predictions of the model M_O_ and the sentiments to compare the development of the sentiment for different organisms. We calculated the mean sentiments over a month and excluded all months that did not have at least 100 tweets for the respective organism. Further, we used the same test as we did for the yearly means to compare the organism class means based on the individual tweet sentiments (positive, negative, and neutral).

Third, we analyzed hashtags as a proxy for the topics a user was talking about in his or her tweet. The hashtag #CRISPR was excluded from the analysis since CRISPR was the overarching topic all tweets had in common. We counted the occurrences of every hashtag per year. We used the exact hashtags and did not group similar hashtags. For example, the hashtags #crisprbaby and #crisprbabies were treated as different hashtags. We did this due to the difficulty of automatically matching similar hashtags, since they can be a composition of multiple words that made strategies like stemming not straightforward. For each hashtag and year, we then calculated the mean sentiment and selected the 15 most common hashtags for each year for further analysis. We then manually compared how these top 15 topics per year increased and decreased in popularity throughout the years, as well as how the sentiments for these topics changed.

In the fourth and last part of our analysis, we based our analysis on the earlier conducted studies. We conducted a literature search in scientific databases according to a predefined search strategy (see Multimedia Appendix 3). The search was conducted in the fall of 2017. We reviewed the resulting studies and identified the reasons why people had a positive or negative attitude towards CRISPR and issues that concerned them. In the process, we summarized these reasons and concerns for each study and compiled a list with a short description for each of them. Since there was thematic overlap across the studies, we inductively determined the themes of these summaries and compiled a regular expression representing each theme based on the summary text. Additionally, we added themes and corresponding regular expressions based on publications and events that occurred between the fall of 2017 and the summer of 2019. The regular expressions then allowed us to automatically check for matches on the entire Twitter dataset as a proxy for the presence of the themes that occurred in the studies. See Multimedia Appendix 4 for the themes and regular expressions.

## Results

### Overview

Our analysis includes over 1,300,000 tweets (dataset P_1_, n=1,311,544) over the time period from January 1, 2013 until May 31, 2019. The predicted sentiments of the tweets were predominantly positive (685,578/1,311,544, 52.3%) or neutral (528,196/1,311,544, 40.3%). Only a minor fraction was predicted as negative (97,770/1,311,544, 7.5%). In the following sections, we report our results focusing on different aspects.

### Temporal Development

**Figure 2:**
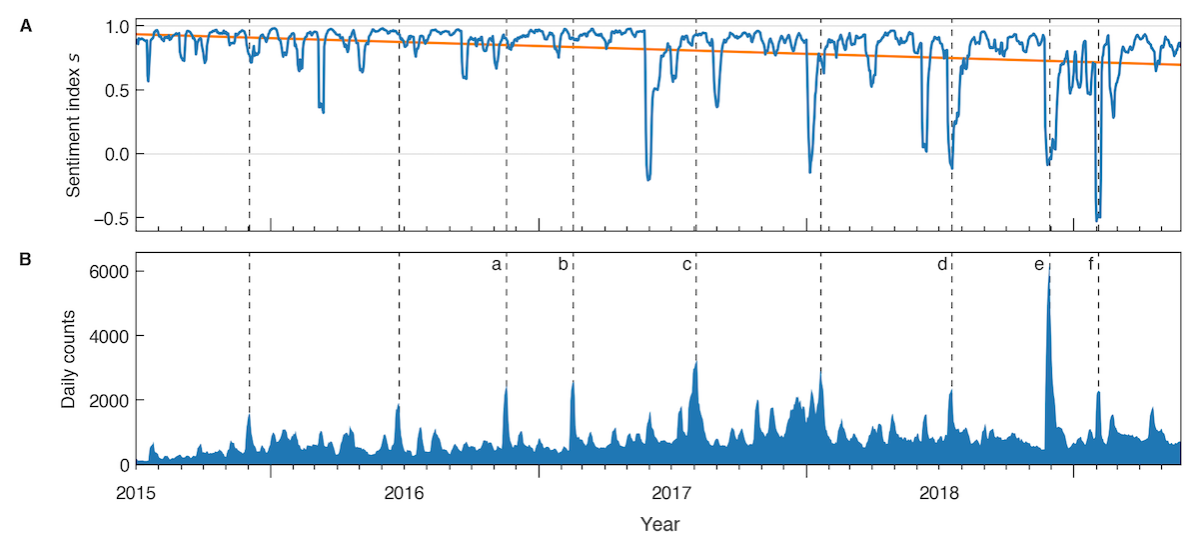
A) Predicted sentiment towards CRISPR between July 2015 and June 2019. The blue curve denotes the sentiment s, which is calculated as the mean of the weighted counts of positive and negative tweets over a centered rolling window of 7 days. The orange curve denotes a linear fit of the sentiment s. B) Daily counts of all analyzed tweets. The blue area shows the daily sum of positive, negative, and neutral tweets as the mean within a 7-day centered rolling window. All peaks above a relative prominence of 0.2 are marked with dashed lines; a-f denote peaks that coincide with certain events.

Figure 2 shows a temporal analysis of the predicted sentiments in relation to key historical events surrounding CRISPR. A sentiment of zero indicates an equal portion of positive and negative tweets, and the values 1 and −1 indicate a signal with only positive or negative tweets, respectively. Figure 2A shows the sentiments between July 2015 and June 2019. The time period before July 2015 was excluded, as activity was too low for a high-resolution sentiment signal. The sentiment remained mostly positive, with an average of 85% positive tweets and only 15% negative tweets. Especially over the initial time period until March 2017, the sentiment shows little variation. After that, the sentiment reveals a series of sharp negative spikes, on multiple occasions dropping below zero. Over the observed time period, the sentiment shows a slight negative trend (slope of −0.061 y^−1^, standard error 0.005 y^−1^), as indicated by the linear trend line in orange. The differences between the yearly means of the tweet sentiments were all significant (*P*<.001; see Multimedia Appendix 5 for all means, standard deviations, and test statistics).

We then compared the sentiment curve to the observed activity surrounding CRISPR in the same time span, as shown in Figure 2B. Shown are the mean daily counts of the sample P_1_ over a sliding window of 7 days. Activity varied considerably, with an average baseline of about 1000 tweets per day and peaks of up to roughly 6000 tweets per day.

We detected 9 peaks of interest. They are marked with dashed lines in Figure 2. When comparing peaks of high activity to the sentiment, it can be seen that peaks of high activity before mid-2018 did not result in a negative sentiment response. Peaks of strong negative sentiment started to appear in 2017 but it was not accompanied by the same level of activity until after 2018.

In a second step, major news events were manually mapped to coinciding peaks (for a full list, see Multimedia Appendix 6). A subset of these peaks was marked with letters a-f in Figure 2B for illustrative purposes. In all cases, the most retweeted tweet within days of the peak was linking a news article describing the event. The events include the first use of CRISPR in humans by a group of Chinese scientists in November 2016 (peak a) and the US Patent Office deciding in favor of the Broad Institute (peak b). Both of these events did not lead to a significant change in sentiment. Peak c coincides with the publication of a study that reported the correction of a mutation in human embryos [57], causing widespread media attention and, as before, did not cause a drop in sentiment. However, in July 2018, a study by the Wellcome Sanger Institute [58] warned about serious side effects, such as cancer, that CRISPR could have when used in humans (peak d). This peak led to a clear negative response in the sentiment index and marks the first negative peak with high media attention. When researcher He Jiankui revealed creating the world’s first genetically edited babies in November 2018 [16] (peak e), the highest activity was recorded. Although He’s revelation caused a strong negative signal, the strongest negative sentiment was recorded shortly after, in February 2019 (peak f). This event coincides with the re-emergence of a news story from August 2017 when biohackers managed to encode a malware program into a strand of DNA [59].

### Organisms

In order to improve our understanding of the sentiment signal, the data were predicted with respect to which organism each tweet was about (see the Methods section). We predicted the organism of the tweets in the dataset P_1_ (n=1,311,544) resulting in the classes animals (7.6%), bacteria (2.4%), embryos (4.3%), humans (30.3%), plants (4.9%), and unspecified (50.6%). It is noteworthy that more than half of all tweets do not specifically refer to an organism in the context of CRISPR. After unspecified*,* the class humans is the second largest group, followed with some margin by animals (eg, mice for animal testing), plants, and embryos*.* The classes humans and embryos combined account for a little more than one-third of all tweets. Tweets specifically mentioning CRISPR in the context of bacteria were rather rare.

**Figure 3:**
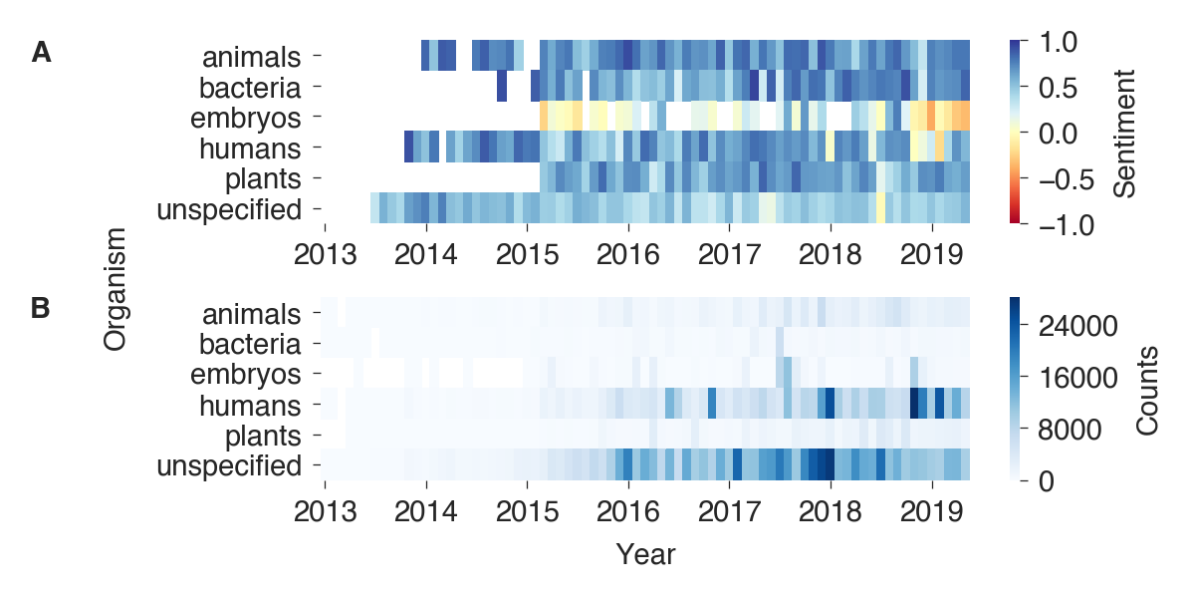
A) Heatmap of monthly sentiments by predicted organism. The sentiments were calculated as the mean of the weighted counts by sentiment (the weights included −1, 0, and 1 for negative, neutral, and positive tweets, respectively) for each month and organism class. Blue and red colors indicate positive and negative sentiment values, respectively. The sentiments of heatmap cells with <100 tweets of that month and organism are transparent. B) Monthly counts by predicted organism.

Figure 3A shows the monthly sentiment for each organism class, which are based on the monthly counts shown in Figure 3B (all monthly means and standard deviations can be found in Multimedia Appendix 7). Of all classes, embryos exhibited the most negative-leaning sentiment (mean sentiment 0.14 over all monthly means) and was also the class with the strongest variations between months (SD 0.27). Further, a relatively high sentiment was measured for the classes animals (mean 0.70, SD 0.14), bacteria (mean 0.65, SD 0.18), and plants (mean 0.61, SD 0.14), followed by the class humans (mean 0.58, SD 0.23), which showed a dip in the sentiment in the months following November 2018. The class unspecified had a slightly lower sentiment (mean 0.45, SD 0.13) compared with the other classes. In addition to this monthly breakdown, the differences between the organism class means based on the individual tweets were all significant (*P*<.001), except for the difference between the class means of bacteria and plants with a 3.8% probability of occurring by chance (*P*=.038; see Multimedia Appendix 5 for all test statistics).

### Hashtags

**Figure 4:**
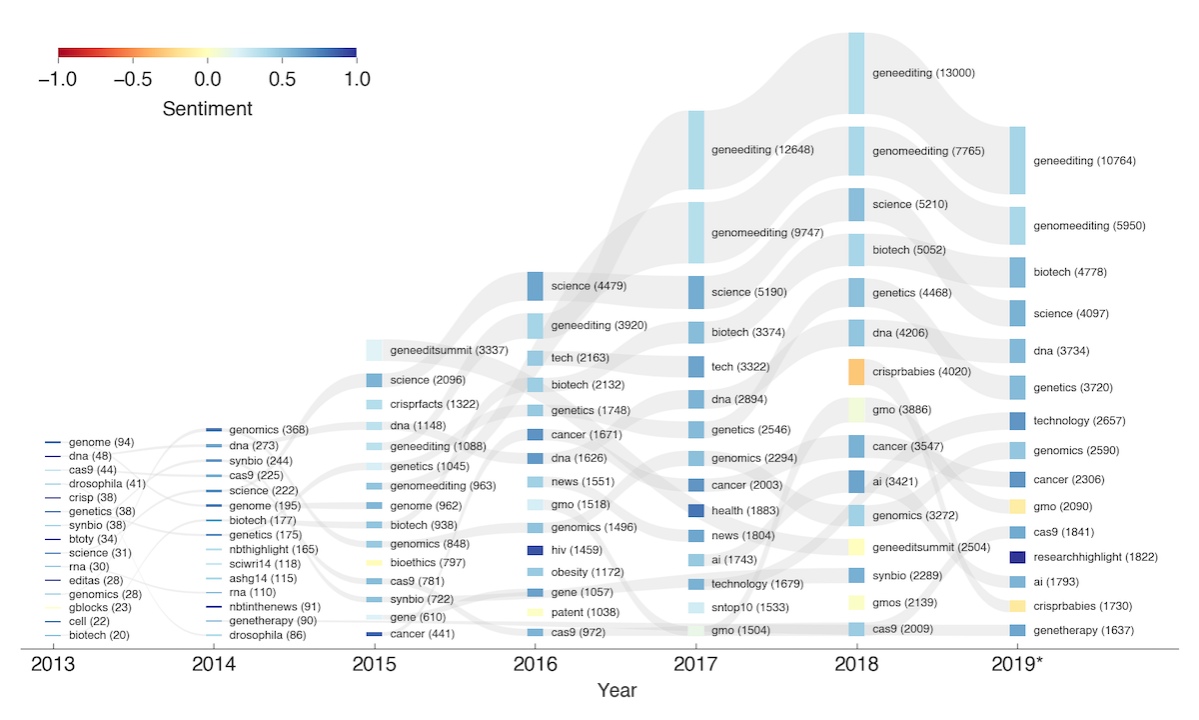
Visualization of the sentiment associated with the most frequently used hashtags every year. For every year, the 15 hashtags with the highest counts for that year are included (the hashtag #crispr was excluded). The hashtags are sorted by yearly counts (indicated by the bar height), where the hashtag with the highest count is at the top. The color represents the average sentiment for the respective hashtag, with blue representing a very positive sentiment and red representing a very negative sentiment. If a hashtag is listed in multiple years, the occurrences are linked with a gray band. The number of tweets with the hashtag is indicated in parentheses next to the respective hashtag. For the year 2019, the counts were extrapolated from the months before June to the full year.

The most frequently used hashtags of every year revealed the topics of highest interest and how they evolved over time (see Figure 4). Naturally, the occurrences of individual hashtags increased over the years along with the total number of tweets. Certain very common hashtags, such as #dna, #science, #biotech, or #geneediting and #genomeediting, appeared as top hashtags in multiple years. When relating the hashtags with the sentiment of the text they appeared in, we can see that most of these common hashtags were used in the context of a positive or very positive sentiment. The 3 hashtags with the most positive sentiments and that were used at least 100 times were #cancer (mean sentiment 0.85, SD 0.36) in 2015, #hiv (mean 0.90, SD 0.34) in 2016, and #researchhighlight (mean 1.00, SD 0.06) in 2019. It is also notable that #science was among the 5 most common hashtags in every year except for 2013 and was consistently related to a positive sentiment, with means between 0.52 (in 2018) and 0.74 (in 2013).

Only a few hashtags were related to negative sentiments. The most prominent one was #crisprbabies, with mean sentiments of −0.30 (SD 0.65) in 2018 and −0.13 (SD 0.63) in 2019, followed by #gmo (mean −0.11, SD 0.76) in 2019, #bioethics (mean −0.02, SD 0.45) in 2015, and #geneeditsummit (mean −0.01, SD 0.46) in 2018. It is worth noting that the hashtag #geneeditsummit only appeared in 2015 and 2018 and that its associated sentiment dropped from 0.20 to −0.01. The hashtag refers to the two summits on human genome editing, which were held in Washington D.C. in 2015 and in Hong Kong in November 2018, coinciding with the first gene editing of viable human embryos. Similarly, the hashtag #gmo became slightly more negative in 2018, with a mean sentiment of 0.09 compared to 2016 (mean 0.24) and 2017 (mean 0.14) and even dropped to −0.11 in 2019. The hashtag #bioethics only appeared in 2015 and was associated with a relatively low sentiment of −0.02. This may highlight the various ethical concerns raised during the 2015 Human Gene Editing summit. See Multimedia Appendix 8 for the full list of the counts, sentiments, and standard deviations of the most used hashtags by year.

### Themes

**Figure 5:**
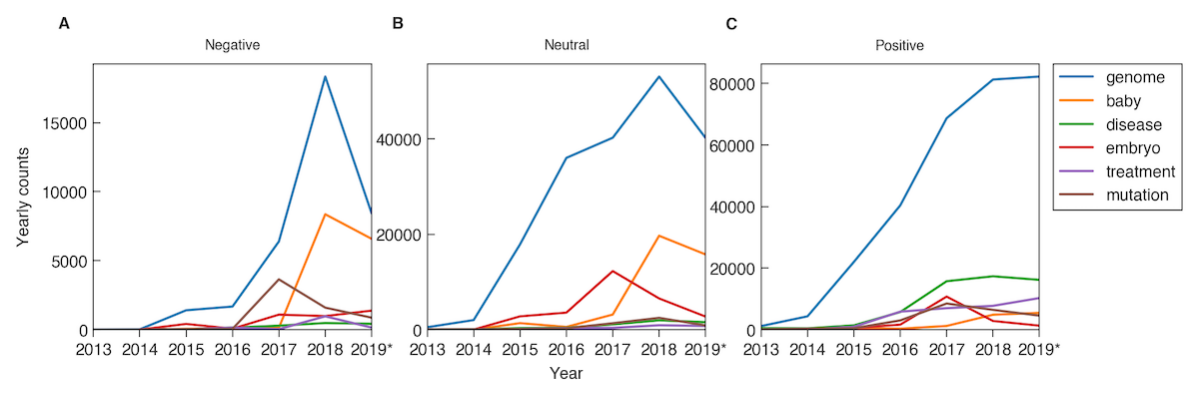
Yearly occurrences of themes. Multiple themes with distinct regex patterns were matched to the text of tweets, and the 6 most frequent themes were selected. Panels A, B, and C show the yearly counts of themes when grouped by negative, neutral, and positive sentiment, respectively. For the year 2019, the counts were extrapolated from the months before June to a full year.

In comparison to the hashtags, the themes derived from previous studies can relate the Twitter discussion to known themes of interest to the public (see the Methods section for a description of the analysis). The 6 themes that were matched most are presented in Figure 5 and grouped by positive, neutral, and negative sentiments. The themes include genome (with a total count of 526,612 [extrapolated for 2019]), baby (68,269), disease (64,181), embryo (49,084), treatment (35,865), and mutation (34,884). Unsurprisingly, the theme “genome” was matched most frequently, occurring in 34% of the tweets.

The reported themes show distinct occurrence patterns depending on sentiment, yielding an aggregated picture of the discussion surrounding CRISPR throughout the years. Spikes are evident in certain years (see Multimedia Appendix 9 for the counts per year of the top 6 themes), and the most significant change in occurrences happened for the theme “baby,” which increased substantially from 2017 to 2018, likely associated with the “CRISPR babies” scandal in November 2018. While a spike could be observed for all 3 sentiments, the increase was far more pronounced in the neutral and negative classes (see Figure 5). The theme “mutation” shows a negative peak in 2017, when risks about potential side effects of CRISPR surfaced. Relative to other themes, the themes “disease” and “treatment” were major themes in a discussion associated with a positive sentiment.

## Discussion

### Principal Findings

We have generated the first high-resolution temporal signal for sentiments towards CRISPR on Twitter, spanning a duration of more than 6 years. Our results suggest that, overall, the CRISPR technology was discussed in a positive light, which aligns well with a previous study that considered the coverage of CRISPR in the press [33]. However, more recently, the sentiment reveals a series of strong negative dips, pointing to a more critical view. The frequency and magnitude of these dips have increased since 2017, which is underlined by the overall declining sentiment. It is noteworthy that the dips usually coincide with high activity, suggesting that many people are only exposed to the topic of CRISPR when it is presented in an unfavorable way.

Further, we could tie the most prominent peaks in tweeting activity to real world events. The last 3 peaks, which coincide with the release of possibly concerning news (side effects, CRISPR babies, malware), also align with strong dips in the tweet sentiment. Together, this indicates that there is at least a partial connection between tweets and the discourse off Twitter and that the sentiment changes are not only the result of a self-contained discussion on the social media platform. Even more so, the peak detection potentially allows the timely identification of significant incidents that can shape public discourse and opinion.

As shown in the breakdown of sentiment by organism, the negative sentiment was stronger in the embryo and human classes but stayed mostly positive towards other organisms. The data therefore suggest that the many ethical issues related to human germline editing are reflected in the tweets. However, criticism may not be targeted at the use of CRISPR in humans per se: Hashtags such as #hiv or #genetherapy were connected to very positive sentiments, which suggests a positive attitude towards developing CRISPR for use in medical treatment. This aspect is further strengthened when considering the sentiment of themes such as “treatment” or “disease.” These observations are in line with several surveys in which participants demonstrated strong support of CRISPR for use in medical treatment but were critical regarding modifications of human germline cells [28-32].

The dataset that includes continuous observations over a long period of time allows for conclusions to be drawn about the public perception of CRISPR both on short and long time scales. For example, when the article on biohacking re-emerged in 2019 (peak f), shortly after the discussions around CRISPR babies, it was discussed in significantly more negative terms than at the time of its publication in 2017. Therefore, the intermediate developments seem to have had a negative influence on the perception of the event. This is in line with the overall negative trend. The presence and absence of themes observed in the data hint at the influence that key events might have on the discussion. While the theme “mutation” was discussed intensely in 2017, its occurrence in tweets dropped in the following year, 2018, in which “baby” became the most occurring theme except for “genome”.

Our results support the use of Twitter and similar platforms for the study of public discourse. Discussion about a subject matter can be investigated in real-time, in depth at the level of individual statements, and on the basis of existing data. The insights gained through such studies can bring new issues to light, indicate which topics need extra attention with respect to ethical considerations and policy making, and allow a quicker response to technological advancements. In addition, the presented method offers a novel approach to promote public engagement, especially in the areas of biotechnologies and health care, as argued by the Nuffield Council on Bioethics [60].

### Limitations

Although the predicted sentiment index seems to overlap well with survey results, it cannot be directly used as a substitute for an opinion poll. Polling allows for the collection of answers to specific questions of interest instead of inferring them from public statements. Furthermore, the Twitter community is not necessarily representative of the whole population of a country. However, sentiment analysis avoids the disadvantages of traditional methods such as response bias and provides more detailed insights through access to granular data of online discussions.

We cannot exclude the possibility that the gradual decrease over time was influenced or caused by a general shift in the sentiment of the scientific Twitter community. Our analysis relies only on Twitter, and we did not validate the findings on another social media platform. Also, we cannot directly tie the sentiment in tweets to the conversation off Twitter. Nonetheless, our results show that there is a connection between tweets, findings in earlier studies, and real-world events and that insights can be gained from this type of analysis on Twitter that are not accessible through other methods.

Further, we acknowledge that most people’s opinions might not fit into the positive, neutral, and negative classes presented in this study. We therefore tried to counteract this problem by categorizing the data not only by sentiment but also by relevance and organism, allowing for a better understanding of the measured sentiment. Furthermore, we recognize the challenging nature of deducing someone’s true opinion based on a short message alone and the fact that it is only possible within a statistical margin of error. This error is slightly larger for the negative class, as the F1 score of this class was relatively low compared to the other classes due to a strong label imbalance. We believe, however, that our method is nevertheless suitable to capture certain trends on a larger scale.

### Conclusions and Future Direction

We demonstrated that the sentiment analysis of tweets provides a high-resolution picture of the ongoing debate on CRISPR, allowing us to study the evolution of the discourse while extending the capacity of traditional methods. Further, the presence of the same themes that have been identified in existing studies confirms the validity of our signal with respect to content. The existence of events that match the activity peaks also indicates the sensitivity of the signal towards off-Twitter incidents. Therefore, our approach offers an additional method to surveys and that can be deployed to get richer information, a larger sample size, and higher temporal resolution.

Future work can go beyond the deduction of sentiments and shed more light on the nature of discussions and arguments raised and how they influence each other, giving a better idea of the reasoning behind people’s opinions. Furthermore, specific topics, such as the discussion surrounding a potential moratorium of CRISPR, may be analyzed in more detail and provide actionable outcomes.

Since the presented analysis can automatically process a large amount of data in almost real-time, it extends the traditional toolset of empirical methods for discourse analysis. It may therefore help analyze public opinion and support policy and decision making.

### Data and Code Availability

The data, machine learning models used, and source code for this analysis can be found in our public repository: https://gitlab.ethz.ch/digitalbioethics/crispr-sentiment-analysis

## Appendix

Multimedia Appendix 1 Yearly counts. Number of tweets per year since January 1, 2013, until May 31, 2019. A steady increase in volume can be observed. In parentheses is the extrapolated number for 2019 (from the first five months).

Multimedia Appendix 2 Model performance. Classification scores for selected models. Subfigures A, B and C correspond to three different classifiers trained for sentiment, relevance and organism, respectively. The y-axis shows the best corresponding model for a specific model type after hyperparameter search was performed. The model types are random (pick a class at random), majority (always pick the most frequent class), bag of words, fastText, BERT and a fine-tuned version of BERT-large (denoted as BERT ft). The x-axis denotes the test performance scores of accuracy (green), and macro-averaged precision (blue), recall (orange) and F1 scores (red). The fine-tuned BERT model was the best performing model for all three classification problems irrespective of the metric used.

Multimedia Appendix 3 Preliminary literature review search strategy and databases.

Multimedia Appendix 4 Themes and regex patterns. Derived themes and corresponding regex patterns from preliminary literature review.

Multimedia Appendix 5 Mean sentiments and test statistics for years and organism classes. On the left, the table shows the mean sentiment (Sent) for each year and organism class based on the sentiments of the individual tweets. Further, the standard deviation (SD) and number (Count) of tweets for each group are reported. On the right, the p-values or the significance level if significant (alpha = 0.001) and the t-values from Welch's t-test among the years and organism class means are shown. A value refers to the comparison between the classes given by its row and column labels. For example, the p-value for Welch's t-test for the difference between the mean of the bacteria class and the plants class is 0.038.

Multimedia Appendix 6 Identified events. Selected events with a peak prominence above 0.2. The marks correspond to the selected events in Figure 2 of the article. Peak times have been automatically detected as described in the methods section. The corresponding events have been inferred from visual inspection of the data.

Multimedia Appendix 7 Monthly mean sentiments and standard deviations per organism. The table shows the mean sentiments (Sent) and their standard deviations (SD) for every month and organism. A dash (–) indicates that less than 100 tweets were in the respective organism class for that month and that we did not calculate the mean sentiment. Months with empty rows had no tweets in that class. The mean values of this table were used in Figure 3.

Multimedia Appendix 8 Top hashtags’ counts and sentiments. List of top 15 hashtags, corresponding counts (Count), sentiments (Sent) and standard deviations (SD) by year. The extrapolated hashtag counts for 2019 are shown under 2019*, the original counts for the first five months under 2019. The mean values of this table were used in Figure 4.

Multimedia Appendix 9 Top themes found in tweets. List of top 6 themes with highest overall occurrence across sentiment. The table shows the number of occurrences in tweets for every sentiment and year. The year 2019 was extrapolated to determine the top themes, indicated by the star (*), based on the first five months of 2019. These counts were used in Figure 5.

## Abbreviations

API: application programming interface
BERT: Bidirectional Encoder Representations from Transformers
BoW: Bag of Words
SVM: support vector machine
