# Supplementary material for: Assessing Public Opinion on CRISPR-Cas9: Combining Crowdsourcing and Deep Learning
Source: J Med Internet Res. 2020 Aug 31;22(8):e17830. doi: 10.2196/17830 (PMC7490675; doi:10.2196/17830)
Supplement: Multimedia Appendix 1 [file jmir_v22i8e17830_app1.pdf]

Multimedia Appendix 1

Year	Number of tweets
2013	4818
2014	20,002
2015	131,211
2016	304,759
2017	437,931
2018	445,744
2019	163,579 (392,590)
**Total**	1,508,044

Table : Yearly counts. Number of tweets per year since January 1, 2013, until May 31, 2019. A steady increase in volume can be observed. In parentheses is the extrapolated number for 2019 (from the first five months).
