# Supplementary material for: Assessing Public Opinion on CRISPR-Cas9: Combining Crowdsourcing and Deep Learning
Source: J Med Internet Res. 2020 Aug 31;22(8):e17830. doi: 10.2196/17830 (PMC7490675; doi:10.2196/17830)
Supplement: Multimedia Appendix 2 [file jmir_v22i8e17830_app2.pdf]

Multimedia Appendix 2

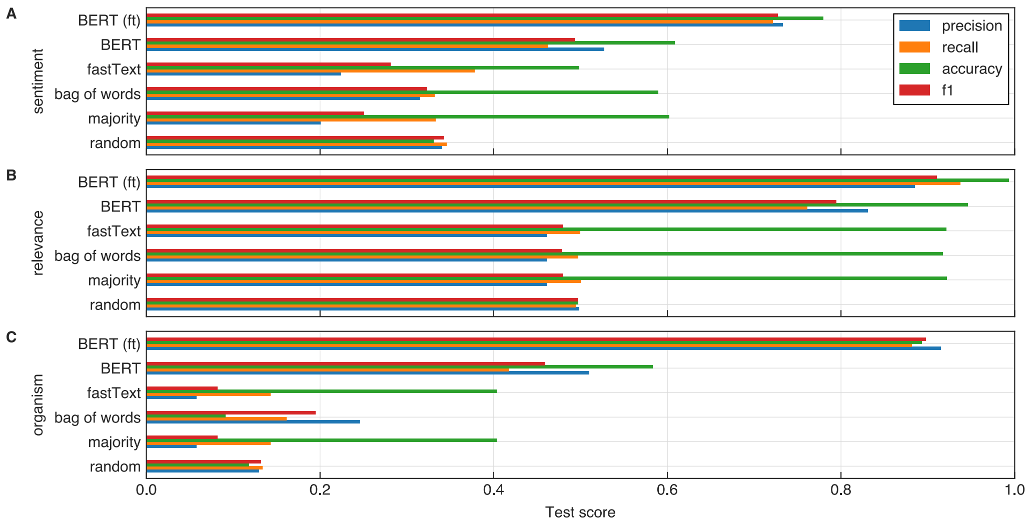

Figure : Model performance. Classification scores for selected models. Subfigures **A**, **B** and **C** correspond to three different classifiers trained for sentiment, relevance and organism, respectively. The y-axis shows the best corresponding model for a specific model type after hyperparameter search was performed. The model types are random (pick a class at random), majority (always pick the most frequent class), bag of words, fastText, BERT and a fine-tuned version of BERT-large (denoted as BERT ft). The x-axis denotes the test performance scores of accuracy (green), and macro-averaged precision (blue), recall (orange) and F1 scores (red). The fine-tuned BERT model was the best performing model for all three classification problems irrespective of the metric used.
