# Supplementary material for: Assessing Public Opinion on CRISPR-Cas9: Combining Crowdsourcing and Deep Learning
Source: J Med Internet Res. 2020 Aug 31;22(8):e17830. doi: 10.2196/17830 (PMC7490675; doi:10.2196/17830)
Supplement: Multimedia Appendix 3 [file jmir_v22i8e17830_app3.pdf]

Multimedia Appendix 3

Preliminary literature review search strategy and databases

Databases used: PubMed, Scopus, Web of science. Matching query in articles' title only:

```
(( crispr OR gene-editing OR "genome editing" ) AND ( attitudes OR opinions OR perspectives OR believes OR reactions OR public ))
```

103 publications were identified by the search (24 PubMed, 41 Scopus, 38 Web of Science). A total of 4 articles were included in the full-text analysis after duplicate removal and exclusion through abstract screening based on exclusion criteria:

- The article is not focussing on CRISPR
- The article is not referring to human subjects
- The article is not considering public opinions/attitudes
- The article is not an empirical study

Resulting documents:

- Blendon, R. J., Gorski, M. T., & Benson, J. M. (2016). The public and the gene-editing revolution. New England Journal of Medicine, 374(15), 1406-1411.
- McCaughey, T., Sanfilippo, P. G., Gooden, G. E., Budden, D. M., Fan, L., Fenwick, E., ... & Liang, H. H. (2016). A global social media survey of attitudes to human genome editing. Cell stem cell, 18(5), 569-572.
- Scheufele, D. A., Xenos, M. A., Howell, E. L., Rose, K. M., Brossard, D., & Hardy, B. W. (2017). US attitudes on human genome editing. Science, 357(6351), 553-554.
- Weisberg, S. M., Badgio, D., & Chatterjee, A. (2017). A CRISPR New World: Attitudes in the Public toward Innovations in Human Genetic Modification. Frontiers in Public Health, 5.
