# Supplementary material for: Assessing Public Opinion on CRISPR-Cas9: Combining Crowdsourcing and Deep Learning
Source: J Med Internet Res. 2020 Aug 31;22(8):e17830. doi: 10.2196/17830 (PMC7490675; doi:10.2196/17830)
Supplement: Multimedia Appendix 4 [file jmir_v22i8e17830_app4.pdf]

Multimedia Appendix 4

<table>
<tr><th>Theme</th><th>Regular expression</th></tr>
<tr><td>disease</td><td>diseases?</td></tr>
<tr><td>health</td><td>restore</td></tr>
<tr><td>therapy</td><td>therapy|therapeutic</td></tr>
<tr><td>germline</td><td>germline|heritable|stem[\s-]cell|heritage</td></tr>
<tr><td>somatic</td><td>somatic</td></tr>
<tr><td>enhancement</td><td>enhanc(e|ement|ing)</td></tr>
<tr><td>improvement</td><td>improv(e|ement|ing)</td></tr>
<tr><td>treatment</td><td>treat(ment|ing)?</td></tr>
<tr><td>reducing</td><td>(lower(ing)?|reduc(e|ing))\s.*risk</td></tr>
<tr><td>prevention</td><td>prevent(ion|ing)?</td></tr>
<tr><td>risk</td><td>risks?</td></tr>
<tr><td>cure</td><td>cur(e|ing)</td></tr>
<tr><td>progress</td><td>scientific progress</td></tr>
<tr><td>traits</td><td>traits?</td></tr>
<tr><td>abilities</td><td>abilit(y|ies)</td></tr>
<tr><td>intelligence</td><td>intelligence</td></tr>
<tr><td>appearance</td><td>appearance</td></tr>
<tr><td>price</td><td>expensive</td></tr>
<tr><td>discovery</td><td>discovery?|anticipat(e|ion)</td></tr>
<tr><td>privacy</td><td>privacy</td></tr>
<tr><td>accuracy</td><td>accuracy</td></tr>
<tr><td>reliability</td><td>reliability</td></tr>
<tr><td>mutation</td><td>mutations?</td></tr>
<tr><td>eugenic</td><td>eugenic</td></tr>
<tr><td>trust</td><td>trust</td></tr>
<tr><td>children</td><td>child(ren)?</td></tr>
<tr><td>genome</td><td>genome|genomics?|genes?|genetic</td></tr>
<tr><td>embryo</td><td>embryo(nic)?</td></tr>
<tr><td>baby</td><td>bab(y|ies)</td></tr>
</table>

Table : Themes and regex patterns. Derived themes and corresponding regex patterns from preliminary literature review.
