# Supplementary material for: Assessing Public Opinion on CRISPR-Cas9: Combining Crowdsourcing and Deep Learning
Source: J Med Internet Res. 2020 Aug 31;22(8):e17830. doi: 10.2196/17830 (PMC7490675; doi:10.2196/17830)
Supplement: Multimedia Appendix 5 [file jmir_v22i8e17830_app5.pdf]

Multimedia Appendix 5

Year	Sent	SD	Count	P_{2013}	P_{2014}	P_{2015}	P_{2016}	P_{2017}	P_{2018}	P_{2019}	T_{2013}	T_{2014}	T_{2015}	T_{2016}	T_{2017}	T_{2018}	T_{2019}
**2013**	0.96	0.29	2495	–	$<.001$	$<.001$	$<.001$	$<.001$	$<.001$	$<.001$	–	3.57	–13.92	–12.33	–19.12	–57.13	–60.08
**2014**	0.98	0.20	10,249		–	$<.001$	$<.001$	$<.001$	$<.001$	$<.001$		–	–37.85	–40.22	–58.51	–143.38	–123.13
**2015**	0.87	0.49	59,840			–	$<.001$	$<.001$	$<.001$	$<.001$			–	5.16	–11.89	–100.74	–90.12
**2016**	0.89	0.46	138,252				–	$<.001$	$<.001$	$<.001$				–	–23.31	–133.50	–106.77
**2017**	0.85	0.53	218,639					–	$<.001$	$<.001$					–	–117.69	–94.49
**2018**	0.62	0.78	253,307						–	$<.001$						–	–12.64
**2019**	0.58	0.81	100,566							–							–

Organism	Sent	SD	Count	P_{animals}	P_{bacteria}	P_{embryos}	P_{humans}	P_{plants}	$P_{\text{unspecified}}$	T_{animals}	T_{bacteria}	T_{embryos}	T_{humans}	T_{plants}	$T_{\text{unspecified}}$
**animals**	0.73	0.50	99,131	–	$<.001$	$<.001$	$<.001$	$<.001$	$<.001$	–	–41.7	–175.18	–145.33	–52.39	–182.95
**bacteria**	0.59	0.51	31,855		–	$<.001$	$<.001$	.038	$<.001$		–	–99.11	–46.78	–2.07	–61.81
**embryos**	0.22	0.58	55,908			–	$<.001$	$<.001$	$<.001$			–	85.75	110.13	74.64
**humans**	0.45	0.70	396,878				–	$<.001$	$<.001$				–	54.34	–29.72
**plants**	0.58	0.57	64,179					–	$<.001$					–	–74.09
**unspecified**	0.41	0.60	663,593						–						–

Table : Mean sentiments and test statistics for years and organism classes. On the left, the table shows the mean sentiment (Sent) for each year and organism class based on the sentiments of the individual tweets. Further, the standard deviation (SD) and number (Count) of tweets for each group are reported. On the right, the p-values or the significance level if significant ($\alpha = 0.001$) and the t-values from Welch's t-test among the years and organism class means are shown. A value refers to the comparison between the classes given by its row and column labels. For example, the p-value for Welch's t-test for the difference between the mean of the bacteria class and the plants class is 0.038.
