# Supplementary material for: Assessing Public Opinion on CRISPR-Cas9: Combining Crowdsourcing and Deep Learning
Source: J Med Internet Res. 2020 Aug 31;22(8):e17830. doi: 10.2196/17830 (PMC7490675; doi:10.2196/17830)
Supplement: Multimedia Appendix 6 [file jmir_v22i8e17830_app6.pdf]

Multimedia Appendix 6

#	Mark	Peak time	Event time	Event	Prominence
1		2015-12-03	2015-12-01	First summit on human gene editing in Washington D.C.	0.21
2		2016-06-24	2016-06-22	U.S. proposal for human trials passes safety reviews	0.26
3	a	2016-11-18	2016-11-15	First time use of CRISPR on humans in China	0.34
4	b	2017-02-17	2017-02-15	Broad Institute prevails in patent conflict	0.33
5	c	2017-08-04	2017-08-02	CRISPR successfully fixes a gene in viable human embryos	0.44
6		2018-01-21	2018-01-19	Study on advances in CRISPR technology	0.37
7	d	2018-07-19	2018-07-16	Study shows the potential for side effects (e.g. deletions) of CRISPR	0.29
8	e	2018-11-29	2018-11-26	”CRISPR babies” scandal	0.97
9	f	2019-02-04	2017-08-10	Biohackers encode a malware program into DNA	0.29

Table : Identified events. Selected events with a peak prominence above 0.2. The marks correspond to the selected events in Figure 2 of the article. Peak times have been automatically detected as described in the methods section. The corresponding events have been inferred from visual inspection of the data.
