# Supplementary material for: Assessing Public Opinion on CRISPR-Cas9: Combining Crowdsourcing and Deep Learning
Source: J Med Internet Res. 2020 Aug 31;22(8):e17830. doi: 10.2196/17830 (PMC7490675; doi:10.2196/17830)
Supplement: Multimedia Appendix 7 [file jmir_v22i8e17830_app7.pdf]

Multimedia Appendix 7

humans

Year	Month	Sent	SD
2013	1	–	–
	2	–	–
	3		
	4	–	–
	5	–	–
	6	–	–
	7	–	–
	8	–	–
	9	–	–
	10	–	–
	11	0.91	0.31
	12	0.63	0.48
2014	1	0.50	0.50
	2	0.79	0.41
	3	–	–
	4	0.64	0.49
	5	0.42	0.51
	6	0.62	0.50
	7	0.71	0.45
	8	0.88	0.33
	9	0.80	0.45
	10	0.64	0.48
	11	0.62	0.50
	12	0.84	0.36
2015	1	0.78	0.45
	2	0.80	0.40
	3	0.49	0.62
	4	0.36	0.57
	5	0.28	0.62
	6	0.57	0.53
	7	0.77	0.44
	8	0.51	0.60
	9	0.43	0.57
	10	0.36	0.57
	11	0.51	0.52
	12	0.23	0.60
2016	1	0.73	0.48
	2	0.47	0.55
	3	0.83	0.39
	4	0.75	0.51
	5	0.48	0.67
	6	0.53	0.53
	7	0.40	0.52
	8	0.77	0.45
	9	0.63	0.53
	10	0.82	0.43
	11	0.50	0.52
	12	0.71	0.48
2017	1	0.59	0.64
	2	0.63	0.51
	3	0.73	0.49
	4	0.81	0.43
	5	0.78	0.50
	6	0.72	0.54
	7	0.64	0.56
	8	0.70	0.53
	9	0.66	0.50
	10	0.72	0.50
	11	0.55	0.58
	12	0.70	0.51
2018	1	0.04	0.81
	2	0.74	0.55
	3	0.68	0.56
	4	0.77	0.47
	5	0.64	0.62
	6	0.13	0.89
	7	0.55	0.66
	8	0.70	0.54
	9	0.69	0.56
	10	0.71	0.51
	11	0.00	0.70
	12	0.10	0.73
2019	1	0.26	0.77
	2	−0.21	0.87
	3	0.40	0.64
	4	0.69	0.56
	5	0.55	0.64

embryos

Year	Month	Sent	SD
2013	1		
	2		
	3		
	4		
	5	–	–
	6		
	7		
	8		
	9		
	10		
	11	–	–
	12	–	–
2014	1		
	2	–	–
	3		
	4		
	5		
	6	–	–
	7		
	8		
	9		
	10		
	11		
	12		
2015	1	–	–
	2	–	–
	3	−0.24	0.50
	4	0.08	0.51
	5	0.03	0.36
	6	−0.03	0.33
	7	−0.15	0.45
	8	–	–
	9	0.05	0.28
	10	−0.01	0.41
	11	–	–
	12	0.05	0.59
2016	1	0.08	0.27
	2	0.22	0.44
	3	–	–
	4	0.34	0.54
	5	0.58	0.55
	6	–	–
	7	–	–
	8	–	–
	9	0.15	0.40
	10	0.16	0.37
	11	0.05	0.22
	12	–	–
2017	1	–	–
	2	0.07	0.27
	3	0.28	0.51
	4	0.18	0.51
	5	–	–
	6	–	–
	7	0.18	0.40
	8	0.61	0.57
	9	0.07	0.71
	10	0.66	0.54
	11	0.15	0.67
	12	0.39	0.49
2018	1	0.34	0.55
	2	–	–
	3	–	–
	4	0.45	0.59
	5	0.35	0.48
	6	0.15	0.39
	7	0.01	0.52
	8	0.54	0.57
	9	0.76	0.44
	10	0.20	0.43
	11	−0.07	0.43
	12	−0.12	0.60
2019	1	−0.41	0.63
	2	−0.03	0.56
	3	−0.14	0.49
	4	−0.29	0.60
	5	−0.35	0.62

animals

Year	Month	Sent	SD
2013	1	–	–
	2	–	–
	3		
	4	–	–
	5	–	–
	6	–	–
	7	–	–
	8	–	–
	9	–	–
	10	–	–
	11	–	–
	12	–	–
2014	1	0.84	0.37
	2	0.50	0.50
	3	0.88	0.33
	4	0.86	0.38
	5	–	–
	6	–	–
	7	0.78	0.43
	8	0.87	0.35
	9	0.72	0.45
	10	0.73	0.44
	11	0.80	0.40
	12	0.43	0.51
2015	1	–	–
	2	–	–
	3	0.74	0.44
	4	0.61	0.50
	5	0.74	0.46
	6	0.79	0.41
	7	0.53	0.54
	8	0.44	0.62
	9	0.59	0.52
	10	0.76	0.44
	11	0.77	0.46
	12	0.83	0.38
2016	1	0.92	0.27
	2	0.81	0.43
	3	0.64	0.50
	4	0.68	0.48
	5	0.84	0.37
	6	0.69	0.48
	7	0.57	0.51
	8	0.77	0.43
	9	0.68	0.50
	10	0.70	0.47
	11	0.62	0.69
	12	0.86	0.37
2017	1	0.48	0.57
	2	0.80	0.44
	3	0.84	0.40
	4	0.58	0.58
	5	0.79	0.49
	6	0.66	0.61
	7	0.50	0.54
	8	0.82	0.41
	9	0.82	0.40
	10	0.84	0.39
	11	0.51	0.64
	12	0.89	0.34
2018	1	0.78	0.46
	2	0.63	0.52
	3	0.64	0.55
	4	0.40	0.53
	5	0.64	0.53
	6	0.78	0.47
	7	0.63	0.57
	8	0.79	0.43
	9	0.84	0.42
	10	0.67	0.60
	11	0.53	0.62
	12	0.32	0.70
2019	1	0.76	0.50
	2	0.70	0.53
	3	0.73	0.55
	4	0.79	0.46
	5	0.79	0.47

bacteria

Year	Month	Sent	SD
**2013**	**1**	–	–
	**2**	–	–
	**3**	–	–
	**4**	–	–
	**5**	–	–
	**6**	–	–
	**7**		
	**8**	–	–
	**9**	–	–
	**10**	–	–
	**11**	–	–
	**12**	–	–
**2014**	**1**	–	–
	**2**	–	–
	**3**	–	–
	**4**	–	–
	**5**	–	–
	**6**	–	–
	**7**	–	–
	**8**	–	–
	**9**	–	–
	**10**	0.91	0.28
	**11**	–	–
	**12**	–	–
**2015**	**1**	–	–
	**2**	0.87	0.33
	**3**	0.74	0.45
	**4**	0.59	0.49
	**5**	0.78	0.42
	**6**	0.51	0.50
	**7**	0.64	0.48
	**8**	–	–
	**9**	0.73	0.46
	**10**	0.56	0.50
	**11**	0.52	0.52
	**12**	0.71	0.46
**2016**	**1**	0.56	0.57
	**2**	0.38	0.49
	**3**	0.48	0.51
	**4**	0.53	0.50
	**5**	0.45	0.50
	**6**	0.62	0.49
	**7**	0.23	0.57
	**8**	0.61	0.49
	**9**	0.65	0.52
	**10**	0.52	0.65
	**11**	0.54	0.50
	**12**	0.58	0.50
**2017**	**1**	0.36	0.49
	**2**	0.64	0.49
	**3**	0.76	0.44
	**4**	0.95	0.23
	**5**	0.27	0.49
	**6**	0.86	0.36
	**7**	0.25	0.44
	**8**	0.75	0.43
	**9**	0.84	0.37
	**10**	0.66	0.48
	**11**	0.81	0.40
	**12**	0.80	0.40
**2018**	**1**	0.49	0.70
	**2**	0.75	0.45
	**3**	0.87	0.35
	**4**	0.75	0.44
	**5**	0.69	0.48
	**6**	0.74	0.48
	**7**	0.78	0.42
	**8**	0.76	0.46
	**9**	0.74	0.44
	**10**	0.91	0.29
	**11**	0.66	0.68
	**12**	0.24	0.56
**2019**	**1**	0.74	0.51
	**2**	0.67	0.47
	**3**	0.70	0.46
	**4**	0.72	0.46
	**5**	0.85	0.38

plants

Year	Month	Sent	SD
**2013**	**1**		
	**2**		
	**3**		
	**4**	–	–
	**5**	–	–
	**6**	–	–
	**7**	–	–
	**8**	–	–
	**9**	–	–
	**10**	–	–
	**11**	–	–
	**12**	–	–
**2014**	**1**	–	–
	**2**	–	–
	**3**	–	–
	**4**	–	–
	**5**	–	–
	**6**	–	–
	**7**	–	–
	**8**	–	–
	**9**	–	–
	**10**	–	–
	**11**	–	–
	**12**	–	–
**2015**	**1**	–	–
	**2**	–	–
	**3**	0.46	0.53
	**4**	0.56	0.53
	**5**	0.73	0.45
	**6**	0.65	0.48
	**7**	0.61	0.49
	**8**	0.42	0.49
	**9**	0.66	0.47
	**10**	0.83	0.39
	**11**	0.61	0.49
	**12**	0.50	0.51
**2016**	**1**	0.71	0.45
	**2**	0.72	0.46
	**3**	0.59	0.51
	**4**	0.26	0.59
	**5**	0.38	0.56
	**6**	0.72	0.45
	**7**	0.48	0.54
	**8**	0.73	0.45
	**9**	0.55	0.53
	**10**	0.44	0.54
	**11**	0.66	0.48
	**12**	0.71	0.46
**2017**	**1**	0.63	0.49
	**2**	0.67	0.47
	**3**	0.75	0.44
	**4**	0.58	0.50
	**5**	0.85	0.37
	**6**	0.70	0.48
	**7**	0.62	0.51
	**8**	0.66	0.47
	**9**	0.84	0.37
	**10**	0.67	0.50
	**11**	0.66	0.55
	**12**	0.64	0.58
**2018**	**1**	0.63	0.53
	**2**	0.68	0.47
	**3**	0.50	0.52
	**4**	0.63	0.52
	**5**	0.73	0.47
	**6**	0.65	0.48
	**7**	0.17	0.70
	**8**	0.34	0.76
	**9**	0.44	0.67
	**10**	0.60	0.59
	**11**	0.47	0.69
	**12**	0.71	0.48
**2019**	**1**	0.69	0.49
	**2**	0.77	0.45
	**3**	0.63	0.52
	**4**	0.59	0.66
	**5**	0.65	0.53

unspecified

Year	Month	Sent	SD
**2013**	**1**	–	–
	**2**	–	–
	**3**	–	–
	**4**	–	–
	**5**	–	–
	**6**	–	–
	**7**	0.31	0.61
	**8**	0.57	0.53
	**9**	0.48	0.51
	**10**	0.40	0.53
	**11**	0.64	0.51
	**12**	0.70	0.47
**2014**	**1**	0.76	0.43
	**2**	0.56	0.50
	**3**	0.75	0.44
	**4**	0.52	0.51
	**5**	0.47	0.52
	**6**	0.61	0.51
	**7**	0.48	0.51
	**8**	0.56	0.52
	**9**	0.52	0.50
	**10**	0.51	0.51
	**11**	0.60	0.50
	**12**	0.37	0.51
**2015**	**1**	0.54	0.51
	**2**	0.58	0.50
	**3**	0.45	0.59
	**4**	0.45	0.55
	**5**	0.38	0.53
	**6**	0.44	0.63
	**7**	0.59	0.55
	**8**	0.55	0.52
	**9**	0.52	0.56
	**10**	0.40	0.54
	**11**	0.49	0.56
	**12**	0.49	0.55
**2016**	**1**	0.40	0.55
	**2**	0.33	0.56
	**3**	0.31	0.64
	**4**	0.49	0.54
	**5**	0.44	0.54
	**6**	0.56	0.52
	**7**	0.31	0.54
	**8**	0.57	0.53
	**9**	0.33	0.58
	**10**	0.33	0.56
	**11**	0.26	0.59
	**12**	0.40	0.53
**2017**	**1**	0.56	0.52
	**2**	0.28	0.49
	**3**	0.46	0.53
	**4**	0.44	0.55
	**5**	0.16	0.72
	**6**	0.12	0.67
	**7**	0.34	0.60
	**8**	0.46	0.57
	**9**	0.43	0.56
	**10**	0.52	0.54
	**11**	0.44	0.57
	**12**	0.38	0.58
**2018**	**1**	0.42	0.60
	**2**	0.50	0.58
	**3**	0.49	0.62
	**4**	0.51	0.63
	**5**	0.51	0.55
	**6**	0.36	0.61
	**7**	−0.03	0.85
	**8**	0.46	0.59
	**9**	0.35	0.59
	**10**	0.49	0.57
	**11**	0.39	0.63
	**12**	0.40	0.64
**2019**	**1**	0.49	0.59
	**2**	0.40	0.60
	**3**	0.45	0.60
	**4**	0.47	0.63
	**5**	0.55	0.56

Table : Monthly mean sentiments and standard deviations per organism. The table shows the mean sentiments (Sent) and their standard deviations (SD) for every month and organism. A dash (–) indicates that less than 100 tweets were in the respective organism class for that month and that we did not calculate the mean sentiment. Months with empty rows had no tweets in that class. The mean values of this table were used in Figure 3.
