# Supplementary material for: Assessing Public Opinion on CRISPR-Cas9: Combining Crowdsourcing and Deep Learning
Source: J Med Internet Res. 2020 Aug 31;22(8):e17830. doi: 10.2196/17830 (PMC7490675; doi:10.2196/17830)
Supplement: Multimedia Appendix 8 [file jmir_v22i8e17830_app8.pdf]

Multimedia Appendix 8

Year	Hashtag	Count	Sent	SD
**2013**	genome	94	0.81	0.40
	dna	48	0.92	0.35
	cas9	44	0.32	0.47
	drosophila	41	0.41	0.50
	crisp	38	1.00	0.00
	genetics	38	0.82	0.39
	synbio	38	0.42	0.64
	btoty	34	1.00	0.00
	science	31	0.74	0.44
	rna	30	0.47	0.51
	editas	28	0.96	0.19
	genomics	28	0.43	0.50
	gblocks	23	0.00	0.00
	cell	22	0.82	0.39
	biotech	20	0.50	0.51
**2014**	genomics	368	0.80	0.40
	dna	273	0.63	0.48
	synbio	244	0.68	0.47
	cas9	225	0.60	0.49
	science	222	0.71	0.46
	genome	195	0.77	0.42
	biotech	177	0.64	0.48
	genetics	175	0.73	0.45
	nbthighlight	165	0.35	0.48
	sciwri14	118	0.29	0.45
	ashg14	115	0.39	0.49
	rna	110	0.53	0.50
	nbtinthenews	91	0.92	0.27
	genetherapy	90	0.54	0.50
	drosophila	86	0.36	0.48
**2015**	geneeditsummit	3337	0.20	0.44
	science	2096	0.56	0.58
	crisprfacts	1322	0.36	0.59
	dna	1148	0.33	0.73
	geneediting	1088	0.34	0.54
	genetics	1045	0.23	0.70
	genomeediting	963	0.49	0.52
	genome	962	0.55	0.59
	biotech	938	0.49	0.53
	genomics	848	0.47	0.55
	bioethics	797	−0.02	0.45
	cas9	781	0.53	0.55
	synbio	722	0.51	0.54
	gene	610	0.21	0.77
	cancer	441	0.85	0.36
**2016**	science	4479	0.62	0.52
	geneediting	3920	0.42	0.53
	tech	2163	0.46	0.59
	biotech	2132	0.45	0.55
	genetics	1748	0.47	0.53
	cancer	1671	0.69	0.46
	dna	1626	0.66	0.50
	news	1551	0.43	0.57
	gmo	1518	0.24	0.57
	genomics	1496	0.50	0.53
	hiv	1459	0.90	0.34
	obesity	1172	0.47	0.58
	gene	1057	0.64	0.51
	patent	1038	0.02	0.28
	cas9	972	0.57	0.52
**2017**	geneediting	12,648	0.38	0.56
	genomeediting	9747	0.36	0.55
	science	5190	0.60	0.56
	biotech	3374	0.53	0.57
	tech	3322	0.63	0.53
	dna	2894	0.56	0.57
	genetics	2546	0.54	0.59
	genomics	2294	0.51	0.57
	cancer	2003	0.68	0.55
	health	1883	0.79	0.49
	news	1804	0.62	0.55
	ai	1743	0.47	0.53
	technology	1679	0.58	0.56
	sntop10	1533	0.25	0.64
	gmo	1504	0.14	0.75
**2018**	geneediting	13,000	0.37	0.63
	genomeediting	7765	0.40	0.58
	science	5210	0.52	0.64
	biotech	5052	0.41	0.60
	genetics	4468	0.52	0.58
	dna	4206	0.49	0.63
	crisprbabies	4020	−0.30	0.65
	gmo	3886	0.09	0.61
	cancer	3547	0.57	0.72
	ai	3421	0.63	0.51
	genomics	3272	0.43	0.60
	geneeditsummit	2504	−0.01	0.46
	synbio	2289	0.58	0.58
	gmos	2139	0.03	0.51
	cas9	2009	0.45	0.65
**2019***	geneediting	10,764	0.42	0.62
	genomeediting	5950	0.40	0.57
	biotech	4778	0.55	0.53
	science	4097	0.57	0.59
	dna	3734	0.54	0.62
	genetics	3720	0.53	0.55
	technology	2657	0.68	0.53
	genomics	2590	0.48	0.55
	cancer	2306	0.68	0.55
	gmo	2090	−0.11	0.76
	cas9	1841	0.61	0.53
	researchhighlight	1822	1.00	0.06
	ai	1793	0.56	0.56
	crisprbabies	1730	−0.13	0.63
	genetherapy	1637	0.62	0.54
**2019**	geneediting	4485	0.42	0.62
	genomeediting	2479	0.40	0.57
	biotech	1991	0.55	0.53
	science	1707	0.57	0.59
	dna	1556	0.54	0.62
	genetics	1550	0.53	0.55
	technology	1107	0.68	0.53
	genomics	1079	0.48	0.55
	cancer	961	0.68	0.55
	gmo	871	−0.11	0.76
	cas9	767	0.61	0.53
	researchhighlight	759	1.00	0.06
	ai	747	0.56	0.56
	crisprbabies	721	−0.13	0.63
	genetherapy	682	0.62	0.54

Table : Top hashtags' counts and sentiments. List of top 15 hashtags, corresponding counts (Count), sentiments (Sent) and standard deviations (SD) by year. The extrapolated hashtag counts for 2019 are shown under 2019*, the original counts for the first five months under 2019. The mean values of this table were used in Figure 4.
