# Supplementary material for: Assessing Public Opinion on CRISPR-Cas9: Combining Crowdsourcing and Deep Learning
Source: J Med Internet Res. 2020 Aug 31;22(8):e17830. doi: 10.2196/17830 (PMC7490675; doi:10.2196/17830)
Supplement: Multimedia Appendix 9 [file jmir_v22i8e17830_app9.pdf]

Multimedia Appendix 9

Sentiment	Year	genome	baby	disease	embryo	treatment	mutation
**negative**	**2013**	8	0	0	0	0	0
	**2014**	24	0	0	1	0	1
	**2015**	1407	58	18	418	16	40
	**2016**	1678	72	149	69	151	58
	**2017**	6392	137	289	1092	35	3647
	**2018**	18,340	8363	484	986	970	1598
	**2019***	8431	6586	425	1382	154	871
	**2019**	3513	2744	177	576	64	363
**neutral**	**2013**	516	0	10	3	5	45
	**2014**	2019	7	41	17	15	89
	**2015**	17,894	1376	331	2798	124	110
	**2016**	36,039	575	403	3579	361	294
	**2017**	40,272	3178	1111	12,293	380	1353
	**2018**	53,096	19,705	1967	6551	932	2485
	**2019***	40,241	15,794	1572	2755	785	898
	**2019**	16,767	6581	655	1148	327	374
**positive**	**2013**	1176	2	492	7	41	31
	**2014**	4347	5	429	15	60	349
	**2015**	22,036	544	1402	570	933	469
	**2016**	40,402	320	5748	1643	5868	3019
	**2017**	68,733	1218	15,754	10,762	6994	8510
	**2018**	81,303	4869	17,358	2828	7757	6450
	**2019***	82,258	5460	16,198	1315	10,284	4567
	**2019**	34,274	2275	6749	548	4285	1903

Table : Top themes found in tweets. List of top 6 themes with highest overall occurrence across sentiment. The table shows the number of occurrences in tweets for every sentiment and year. The year 2019 was extrapolated to determine the top themes, indicated by the star (*), based on the first five months of 2019. These counts were used in Figure 5.
